# Acne Necrotica in a Woman With Systemic Lupus Erythematosus and Systemic Sclerosis

**DOI:** 10.7759/cureus.23008

**Published:** 2022-03-09

**Authors:** Alejandro Barrera-Godínez, Ana Fernanda Oliveros-Hernández, Michelle Gatica-Torres, Braulio Martínez-Benitez, Judith Dominguez-Cherit

**Affiliations:** 1 Dermatology, Instituto Nacional de Ciencias Médicas y Nutrición Salvador Zubirán, Mexico City, MEX; 2 Internal Medicine, Hospital Ángeles Mocel, Mexico City, MEX; 3 Pathology, Instituto Nacional de Ciencias Médicas y Nutrición Salvador Zubirán, Mexico City, MEX

**Keywords:** systemic lupus erythematosus, systemic sclerosis, varioliform, varioliform scars, acne necrotica varioliform, necrotizing lymphocytic folliculitis

## Abstract

We report the case of a 25-year-old woman who presented to the outpatient dermatology clinic with a history of systemic lupus erythematosus, systemic sclerosis, and primary hypothyroidism. She complained of a one-year history of cutaneous lesions that were pruriginous and evolved into crusts and weeks later resolved with varioliform scarring. Clinicopathological correlation established a diagnosis of acne necrotica varioliformis. This report highlights the clues and pitfalls in its diagnosis and reviews associated systemic diseases.

## Introduction

Acne necrotica is a follicular disorder described as bizarre and enigmatic [1]. Its rarity and a brief mentioned in fundamental textbooks make it an under-recognized entity representing a diagnostic challenge because of its unique clinicopathological characteristics. This report highlights the clues and pitfalls in its diagnosis and reviews associated systemic diseases.

## Case presentation

A 25-year-old woman presented to the outpatient dermatology clinic with a history of systemic lupus erythematosus (SLE), limited systemic sclerosis (SS), and primary hypothyroidism. SLE diagnosis was made eight years before, and past systemic activity included arthritis, thrombocytopenia, diffuse alveolar hemorrhage, and mononeuritis multiplex. There was no history of cutaneous lupus. SS was present for two years and featured Raynaud's phenomenon, digital tip ulcers, and sclerodactyly. Calcinosis cutis was not documented. Medical treatment for these conditions consisted of prednisone, azathioprine, hydroxychloroquine, and levothyroxine. A copper intrauterine device served as a birth control method.

**Figure 1:**
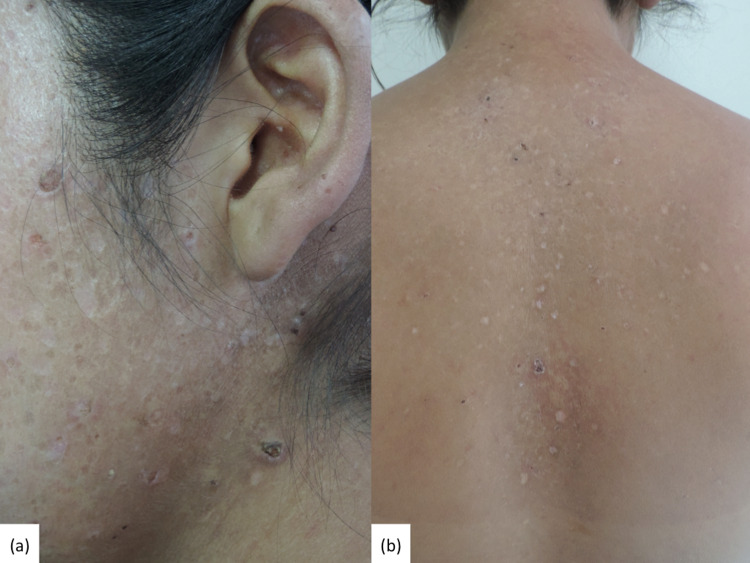
Clinical findings in acne necrotica Panel (a) highlights the concomitant findings of umbilicated papules with a central crust and scattered irregular varioliform scars. Panel (b) shows the disease extended to involve the central back.

She complained of a one-year history of cutaneous lesions that were pruriginous and evolved into crusts and weeks later resolved with scarring. They first appeared on the chest and back and developed on the face eight months later. After six weeks, treatment with adapalene (gel, 0.3%) showed no improvement. Skin lesions predominated on the head (forehead, periorbital, nasal, and auricular), central chest, arms, and back (Figure 1). The most striking finding was the presence of discrete, irregular, varioliform scars that measured 2 to 3 mm.

**Figure 2:**
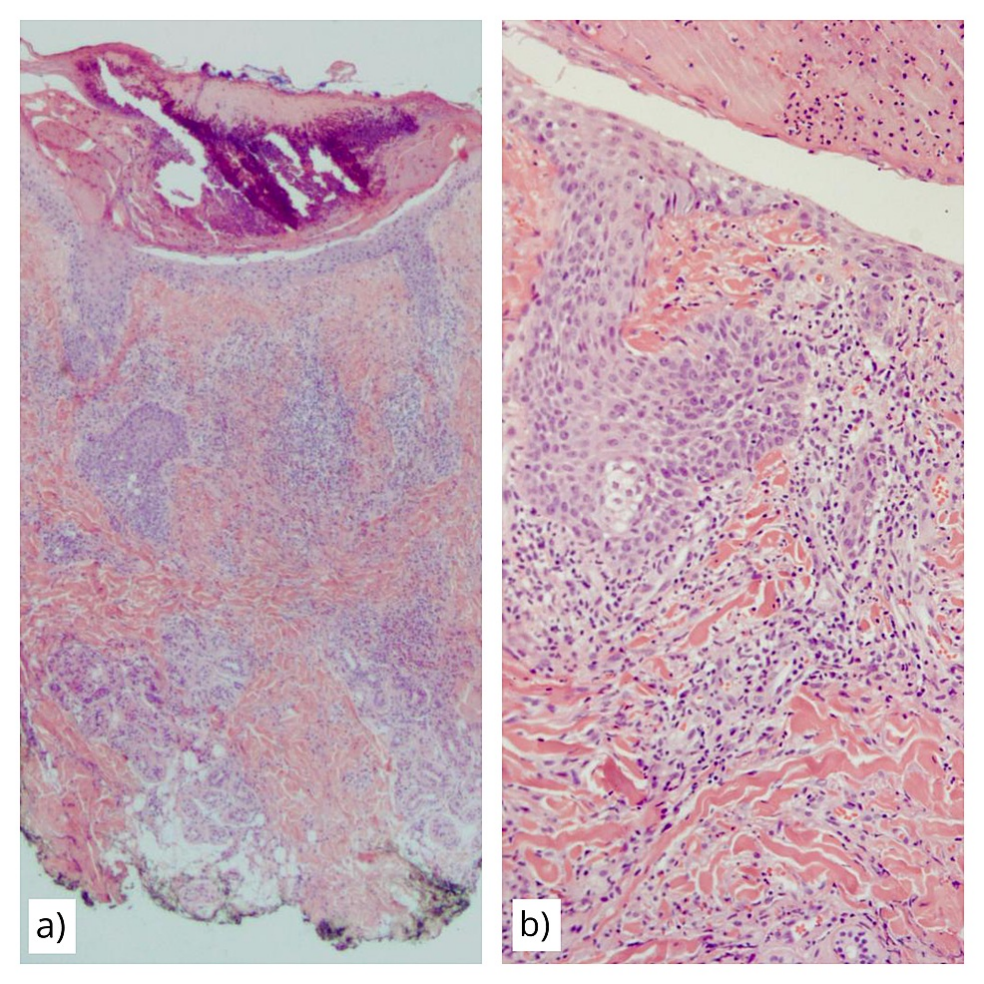
Histopathological findings in acne necrotica. Panel (a) shows a low-power view (H&E, 10x) featuring a central crust with epidermal and follicular necrosis with a perivascular and perifollicular inflammatory infiltrate. Panel (b) showcases the dense, mixed, perifollicular infiltrate (H&E, 40x).

Erythematous papules with a central serosanguineous crust were less frequently observed. A punch biopsy was performed on a crusted papule on the forehead. Histopathology showed epidermal necrosis centered on a follicular unit accompanied by a mixed inflammatory infiltrate composed mainly of lymphocytes with prominent eosinophils and occasional neutrophils (Figure 2). Clinicopathological correlation established a diagnosis of necrotizing lymphocytic folliculitis. Microbiological culture studies were not obtained. Treatment was begun with doxycycline 100 mg twice daily, but the patient had poor adherence due to gastrointestinal upset. One month after the biopsy, the patient developed pneumonia that progressed to septic shock and acute kidney injury that required mechanical ventilation and renal replacement therapy. She rejected invasive maneuvers and unfortunately expired during the following days.

## Discussion

Acne necrotica is an inflammatory disorder of unknown etiology centered on the hair follicle that results in necrosis of the follicular epithelium and adjacent skin. It is also known as necrotizing lymphocytic folliculitis because of the histopathological findings of early lesions [1]. It represents approximately 1% of all biopsies of cicatricial alopecias [2]. However, alopecia is not a part of its clinical presentation, and this clinicopathological dissociation contributes to its underrecognition. Lesions begin as small, erythematous, follicular papules that are subtle and easily missed [3]. In this stage, histopathology features the characteristic perifollicular lymphohistiocytic infiltrate accompanied by spongiosis and keratinocyte necrosis within the external root sheath. The follicular papules develop umbilication and crusting secondary to the underlying necrosis in a few days. Biopsy findings in more evolved lesions are nonspecific, as seen in this case, and include a dense, mixed, inflammatory infiltrate around a necrotic follicular unit surrounded by epidermal and dermal necrosis. Clinicopathological correlation is essential for biopsies from older lesions because of the brevity of characteristic findings [3]. Over the following weeks, the crusts are sloughed, and punched-out varioliform scars remain. These scars represent a significant clinical clue to guide the diagnosis since early lesions are subtle or easily excoriated. Few entities featuring varioliform scars include varicella, pityriasis lichenoides et varioliformis acuta (PLEVA), malignant atrophic papulosis, atrophia maculosa varioliformis cutis, and acne necrotica [4]. Apart from the scarring, other essential clues to distinguish from acneiform eruptions include pruritus and scalp involvement. 

No reports analyze the association between acne necrotica and systemic diseases: existing papers inconsistently describe comorbidities rheumatoid arthritis, osteoarthritis [5], sacroiliitis, and pulmonary tuberculosis [6]. Our case features coexistence with two autoimmune diseases. The occurrence of acne necrotica in the setting of a mixed group of conditions could represent a coincidental event. Further research is required to determine if an association is plausible.

## Conclusions

Acne necrotica is a briefly explored entity that can represent a diagnostic challenge. Essential keys to identify it include the presence of varioliform scarring, the extension to the scalp, and the associated pruritus. Additional studies are needed to establish an association with systemic diseases.
